# Retrospective Analysis of Nontuberculous Mycobacterial Infection and Monochloramine Disinfection of Municipal Drinking Water in Michigan

**DOI:** 10.1128/mSphere.00160-19

**Published:** 2019-07-03

**Authors:** Nadine Kotlarz, Lutgarde Raskin, Madsen Zimbric, Josh Errickson, John J. LiPuma, Lindsay J. Caverly

**Affiliations:** a Department of Civil and Environmental Engineering, University of Michigan, Ann Arbor, Michigan, USA; b Department of Pediatrics, University of Michigan Medical School, Ann Arbor, Michigan, USA; c Consulting for Statistics, Computing and Analytics Research (CSCAR), University of Michigan, Ann Arbor, Michigan, USA; University of Iowa

**Keywords:** disinfection, drinking water, infection, monochloramine, nontuberculous mycobacteria

## Abstract

Infections by nontuberculous mycobacteria (NTM) are primarily acquired from environmental sources, including exposure to municipally treated drinking water. Higher levels of NTM have been reported in drinking water disinfected with monochloramine than in that disinfected with chlorine. However, the relationships between water treatment practices and NTM infection are unclear. The objective of this study was to examine a possible relationship between residual disinfectant used for municipal drinking water treatment (monochloramine or chlorine) and NTM infection. We retrospectively reviewed NTM diagnostic tests performed at a single health care center during a 15-year period. Information on municipal water treatment practices, including disinfectant and primary source water type, was obtained for 140 cities. Based on a logistic regression model, municipal drinking water disinfection with monochloramine compared to chlorine was not associated with NTM infection (*P = *0.24). An additional model variable examining water source showed that the likelihood of having an NTM infection was 1.46 times higher for patients residing in cities with drinking water derived from surface water than for those residing in cities with drinking water derived from groundwater (odds ratio [OR], 1.46; 95% confidence interval [CI], 1.03 to 2.08; *P = *0.04). In an inverse propensity score weighted regression, monochloramine disinfection was also not associated with NTM infection. A moderate effect on NTM infection rates was observed in the weighted regression for municipal drinking water derived from surface water, though the results were not statistically significant (OR, 1.24; 95% CI, 0.92 to 1.69; *P = *0.17).

**IMPORTANCE** Infections by nontuberculous mycobacteria (NTM) result in significant morbidity, mortality, and health care costs. NTM are primarily acquired from environmental sources, including exposure to municipally treated drinking water. Higher levels of NTM have been reported in drinking water disinfected with monochloramine than in drinking water disinfected with chlorine. Our results suggest that municipal drinking water disinfection with monochloramine compared to chlorine is not associated with higher risk of NTM infection. This is important given that regulations that limit drinking water concentrations of disinfection by-products, which are formed primarily when chlorine disinfection is used, incentivize drinking water utilities to change from chlorine disinfection to monochloramine disinfection.

## Abstract (precis)

Infections by nontuberculous mycobacteria (NTM) result in significant morbidity, mortality, and health care costs. NTM are primarily acquired from environmental sources, including exposure to municipally treated drinking water. Higher levels of NTM have been reported in drinking water disinfected with monochloramine than in drinking water disinfected with chlorine. Our results suggest that municipal drinking water disinfection with monochloramine compared to chlorine is not associated with higher risk of NTM infection. This is important given that regulations that limit drinking water concentrations of disinfection by-products, which are formed primarily when chlorine disinfection is used, incentivize drinking water utilities to change from chlorine disinfection to monochloramine disinfection.

## INTRODUCTION

Each year, an estimated 16,000 hospitalizations, generating $425 million in costs, and 3,000 deaths are attributed to infections by nontuberculous mycobacteria (NTM) in the United States (1, 2). NTM infections are acquired primarily from waterborne sources, including municipally treated drinking water (2 – 4). NTM have been detected throughout drinking water treatment systems, including in natural water bodies used as sources for drinking water production (5), at treatment plants (5, 6), and in water distribution systems and potable water taps (5, 7, 8).

Disinfection of water supplies at treatment plants involves two stages: primary disinfection to inactivate microorganisms coming into the treatment plant and residual or secondary disinfection to limit microbial growth in the water distribution system. Free chlorine is the most common drinking water disinfectant used in the United States, followed by monochloramine, a disinfectant formed when ammonia and free chlorine react in water. Monochloramine is an attractive choice for residual disinfection in that it reacts more slowly with biomolecules than chlorine (9), allowing its concentration to be maintained longer at a level sufficiently high for microbial inactivation. Additionally, monochloramine produces fewer of the disinfection by-products (e.g., trihalomethanes) regulated by the U.S. Environmental Protection Agency (EPA) (10).

NTM are relatively resistant to common water disinfection procedures (11, 12). Several studies of full-scale and simulated drinking water systems have identified higher abundances of NTM in water disinfected with monochloramine than in water disinfected with chlorine (8, 13 – 18). The impact of monochloramine disinfection on the risk of NTM infection, however, is unknown. The objective of this study was to test relationships between the secondary disinfectant used for municipal drinking water treatment (monochloramine or chlorine) and NTM infection.

## RESULTS

**FIG 1:**
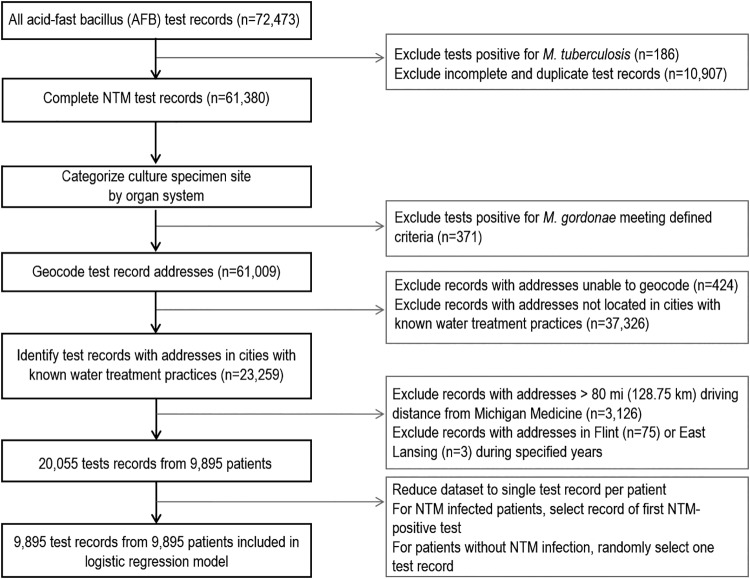
Schematic of inclusion and exclusion criteria for selection of the study cohort.

We retrospectively reviewed 72,473 mycobacterial diagnostic tests performed over a 15-year period from 31,696 patients at Michigan Medicine, the University of Michigan’s academic medical center. After filtering the data for our defined exclusion criteria, 9,895 patients and test records were identified for inclusion in a logistic regression analysis (Fig. 1). Of these, 468 patients (4.7%) had at least one NTM-positive test result. Demographic and clinical characteristics of patients included in the study are shown in Table 1. NTM were most commonly isolated from a pulmonary source, and Mycobacterium avium complex was the most common NTM identified.

**TABLE 1:** Patient demographic and clinical characteristics

Characteristic	Value(s)	
	NTM-positive patients (*n* = 468) (%)	NTM-negative patients (*n* = 9,427) (%)
Age, mean ± SD	54.5 ± 20.7	50.5 ± 21.7
Sex		
Female	240 (51.3)	4,546 (48.2)
Male	228 (48.7)	4,881 (51.8)

Predisposed^*a*^	255 (54.5)	2,805 (29.8)

NTM test site		
Lung	310 (66.3)	2,990 (31.7)
Blood and cardiovascular	13 (2.8)	490 (5.2)
Genitourinary	6 (1.3)	79 (0.8)
Skin and musculoskeletal	6 (1.3)	753 (8)
Gastrointestinal	3 (0.6)	516 (5.5)
Sinus	3 (0.6)	45 (0.5)
Lymphatic	1 (0.2)	47 (0.5)
Central nervous system	0	360 (3.8)
Other	47 (10.0)	2,489 (26.4)
Missing/unknown	79 (16.9)	1,658 (17.6)
NTM species		
M. avium complex	213 (45.5)	
M. chelonae/M. abscessus complex^*b*^	69 (14.7)	
M. fortuitum	42 (9.0)	
M. terrae	36 (7.7)	
M. mucogenicum	20 (4.3)	
M. gordonae	16 (3.4)	
M. kansasii	15 (3.2)	
Other	14 (3.0)	
Unknown/missing	43 (9.2)	

a Defined using ICD-9 and ICD-10 diagnosis codes for immunocompromising conditions and structural lung disease that may predispose to NTM infection (2, 31 – 34) (Table S1).

b The clinical microbiology laboratory did not differentiate these species during part of the study period.

10.1128/mSphere.00160-19.1 TABLE S1 340 ICD codes for predisposing conditions for NTM infection. Download Table S1, DOCX file, 0.04 MB. Copyright © 2019 Kotlarz et al. 2019 Kotlarz et al. This content is distributed under the terms of the Creative Commons Attribution 4.0 International license.

Data on municipal drinking water (i.e., the type of secondary disinfectant [chlorine or monochloramine]) and source water [surface or groundwater]) were obtained for 140 cities in Michigan (Table S2). Three cities (Ann Arbor, East Lansing, and Lansing) used monochloramine for secondary disinfection, and the rest used chlorine. The majority of cities (98 cities; 70%) derived their drinking water primarily from surface water, and the rest used groundwater. The majority of patients (83.9%) had home addresses in cities that derived drinking water from surface water, and the majority (77.6%) had home addresses in cities that used chlorine disinfection (Table 2).

**TABLE 2:** Distribution of municipal drinking water disinfectant and source water type for patients’ home addresses

Parameter	Values		
	Total no. (%) of patients (*n* = 9,895)	No. (%) of NTM- negative patients (*n* = 9,427)	No. (%) of NTM- positive patients (*n* = 468)
Disinfectant			
Chlorine	7,682 (77.6)	7,338 (77.8)	344 (73.5)
Monochloramine	2,213 (22.4)	2,089 (22.2)	124 (26.5)

Source water type			
Surface water	8,301 (83.9)	7,882 (83.6)	419 (89.5)
Groundwater	1,594 (16.1)	1,545 (16.4)	49 (10.5)

10.1128/mSphere.00160-19.2 TABLE S2 Primary source water (SW, surface water; GW, groundwater) and disinfectant (monochloramine or chlorine) for 140 cities in Michigan. Download Table S2, DOCX file, 0.03 MB. Copyright © 2019 Kotlarz et al. 2019 Kotlarz et al. This content is distributed under the terms of the Creative Commons Attribution 4.0 International license.

In a logistic regression analysis of patient and city variables as predictors of NTM infection, significant predictors included diagnosis for a predisposing condition (odds ratio [OR], 6.67; 95% confidence interval [CI], 3.88 to 11.51; *P < *0.001) and age in years (OR, 1.01; 95% CI, 1.01 to 1.02; *P < *0.001) (Table 3). The interaction between age and predisposing condition was statistically significant (*P = *0.002), and inclusion of this interaction improved the model Akaike information criterion (AIC). Although the type of disinfectant used did not contribute significantly (*P = *0.24), the use of surface water as the primary drinking water source was a significant predictor of NTM infection (OR, 1.46; 95% CI, 1.03 to 2.08; *P = *0.04). An interaction between surface water and monochloramine was added to the model, but the results of the addition were not statistically significant (*P = *0.75) and did not improve the model AIC; therefore, the interaction was not included. The following patient variables were included in the model but did not yield statistically significant results: sex, sample collection year, and driving distance from home address to Michigan Medicine. The following city variables were also included in the model but did not yield statistically significant results: population density, percent population that was white, percent population older than 65 years, and median household income.

**TABLE 3:** Results of logistic regression analysis^*a*^

Predictor	Variable type	*P* value	OR (95% CI)
Sex (male)	Patient	0.06	0.84 (0.69–1.01)
Age (yrs)	Patient	<0.001	1.01 (1.01–1.02)
Predisposed	Patient	<0.001	6.67 (3.88–11.51)
Interaction between age and predisposing condition	Patient	0.002	0.99 (0.98–0.99)
Sample yr	Patient	0.07	0.98 (0.96–1.00)
Driving distance to Michigan Medicine	Patient	0.69	1.00 (1.00–1.00)
Population density	City	0.06	1.00 (1.00–1.00)
Drinking water source (surface water)	City	0.04	1.46 (1.03–2.08)
Drinking water disinfectant (monochloramine)	City	0.24	1.22 (0.87–1.68)
% population older than 65 yrs	City	0.64	0.99 (0.96–1.03)
% population white	City	0.06	1.01 (1.00–1.01)
Log(median income)	City	0.44	1.18 (0.77–1.80)

a Significant predictors were based on α = 0.05.

This study used observational data from an academic medical center. Because the assignment of patients to a municipal drinking water treatment system (chloraminated versus chlorinated water) was not random, the estimation of the effect of disinfectant type on NTM infection might be biased by an imbalance in covariates between patients in cities using monochloramine disinfection and patients in cities using chlorine disinfection. To address this potential imbalance, we estimated each patient’s propensity to have water with monochloramine disinfection and used these estimated propensities to generate inverse propensity score weights. The balance improved for most covariates based on smaller differences in mean values between the monochloramine and chlorine groups for the weighted data (Table S3). In a weighted logistic regression analysis, disinfectant type was still not associated with a significant difference in NTM infection. The effect of municipal water derived from surface water on NTM infection decreased compared with the unweighted model, and the effect was no longer statistically significant (OR, 1.24; 95% CI, 0.92 to 1.69; *P = *0.17), although the direction of the effect (associating surface water with NTM infection) was consistent with the initial analysis (Table S4).

10.1128/mSphere.00160-19.3 TABLE S3 Differences between mean values and *P* values for monochloramine and chlorine groups for unweighted and weighted data. Download Table S3, DOCX file, 0.02 MB. Copyright © 2019 Kotlarz et al. 2019 Kotlarz et al. This content is distributed under the terms of the Creative Commons Attribution 4.0 International license.

10.1128/mSphere.00160-19.4 TABLE S4 Inverse propensity score weighted regression *P* values and odds ratios. Download Table S4, DOCX file, 0.02 MB. Copyright © 2019 Kotlarz et al. 2019 Kotlarz et al. This content is distributed under the terms of the Creative Commons Attribution 4.0 International license.

## DISCUSSION

Regulations that limit drinking water concentrations of disinfection by-products, which are formed primarily when chlorine disinfection is used, have incentivized drinking water utilities to convert from chlorine to monochloramine disinfection (19, 20). A possible relationship between monochloramine disinfection of drinking water and NTM infection risk had not been investigated previously, though higher NTM concentrations are frequently recovered from chloraminated drinking water than from chlorinated water (8, 13 – 18). Our finding that monochloramine disinfection was not associated with an increased risk of NTM infection will need to be confirmed by other studies in geographic regions where the proportion of municipalities using monochloramine is higher (e.g., in the southeastern United States) and/or in relation to health care-related outbreaks (21, 22). Municipal water authorities in cities using surface water with higher concentrations of organic carbon, bromide, or nitrogen tend to use monochloramine rather than chlorine to minimize formation of regulated disinfection by-products (10). It is therefore possible that the higher NTM concentrations reported in chloraminated water are related to poorer quality of the source water, including higher bacterial concentrations, rather than to the disinfectant type. Consistent with this hypothesis, the source waters for the three cities in this study that used monochloramine were rated by the U.S. EPA as having high susceptibility to contamination (23 – 25).

By analyzing additional city-level variables included in our model, we determined that the likelihood of having an NTM infection was 1.46 times higher for a patient residing in a city with drinking water derived from surface water than for a patient living in a city using groundwater as its drinking water source. While the inverse propensity score weighted regression analysis resulted in a reduction of the magnitude of this effect and loss of statistical significance, the odds of having an NTM infection being 1.24 times higher for patients living in cities with surface water sources found in the weighted analysis may still represent a large practical effect. The association between NTM infection and surface water is consistent with prior epidemiologic studies, which reported associations between higher prevalence rates of NTM infection and surface water exposure through closer household proximity to surface water (26) and residence in certain watersheds (27). Further evaluation of a relationship between surface water-derived drinking water and NTM infection risk is needed, including determination of differences in NTM abundance and characterization of NTM species (pathogenic versus nonpathogenic) in surface water relative to groundwater.

This study included NTM diagnostic test records from a single academic medical center in Michigan. As NTM infection is not required to be reported in Michigan, comprehensive data on NTM infections were not available for the entire state. We were thus unable to use the general population as a comparison group for our study of the effect of monochloramine disinfection on NTM infection risk. Instead, we compared NTM-positive patients with NTM-negative patients within a single medical center. This study of NTM infection risk among patients tested for NTM infection (i.e, suspected to have NTM infection) has certain limitations. First, we assumed that the likelihood of a doctor suspecting an NTM infection and ordering an acid-fast bacillus (AFB) culture is independent of the type of municipal water disinfectant used for a patient’s home water supply. Another limitation of analyzing test records from patients suspected to have NTM infection is that we cannot use these data to determine the prevalence of NTM infection, as this would require NTM infection data for the entire state. To reduce referral bias related to the distance of each patient’s home from Michigan Medicine, the analysis was limited to records with addresses within 80 miles (128.75 km) driving distance of Michigan Medicine, and driving distance from home addresses to Michigan Medicine was included as a covariate in the model.

While we focused our study on municipal drinking water supply and disinfectant type, NTM infections may be acquired from sources unrelated to home drinking water supplies, including soil and other water-exposed surfaces outside the home (22, 28). Additionally, all addresses located within a city’s geographic limits were assumed to be serviced by that city’s municipal drinking water system. Some addresses within city limits may rely on private wells for drinking water, resulting in misclassification. We estimate that this occurred in less than 5% of the addresses for larger cities based on an analysis that showed that 1.8% of homes located within the city limits for Ann Arbor, MI, do not receive municipal drinking water (T. Baughman, personal communication, 17 April 2019).

In summary, municipal drinking water disinfectant type (chlorine versus monochloramine) was not significantly associated with NTM infection. This is important given that regulations that limit drinking water concentrations of disinfection by-products, which are formed primarily when chlorine disinfection is used, incentivize drinking water utilities to change from chlorine to monochloramine disinfection.

## MATERIALS AND METHODS

### NTM test records.

A retrospective study of patients tested for mycobacterial infection through the course of routine clinical care at Michigan Medicine was carried out. Records of all AFB smears and cultures performed at the Michigan Medicine clinical microbiology laboratory from January 2000 through September 2015 were obtained with the approval of the University of Michigan’s Institutional Review Board. For each test record, the following clinical data were obtained using University of Michigan’s DataDirect self-service data tool (29): patient sex, age, and home address at the time of sample collection, associated International Classification of Diseases (ICD) (9th and 10th revisions, clinical modification [ICD-9-CM and ICD-10-CM]) diagnosis codes, sample source, and AFB smear and culture results. NTM species identification was performed by the Michigan Department of Community Health from 2000 through April 2014. Since May 2014, NTM species identification has been performed by the Michigan Medicine clinical laboratory using matrix-assisted laser desorption ionization–time of flight mass spectrometry. Specimen sources for NTM-positive cultures were categorized by organ system as “lung,” “blood and cardiovascular,” “skin and musculoskeletal,” “gastrointestinal,” “genitourinary,” “sinus,” “lymphatic,” “central nervous system,” “other,” or “missing/unknown” (Table S5).

10.1128/mSphere.00160-19.5 TABLE S5 Terms used for grouping NTM specimen collection sites into organ system categories. Download Table S5, DOCX file, 0.02 MB. Copyright © 2019 Kotlarz et al. 2019 Kotlarz et al. This content is distributed under the terms of the Creative Commons Attribution 4.0 International license.

Patients were categorized as NTM positive if they had either an AFB culture positive or an AFB smear positive with an inconclusive culture result (e.g., NTM species not identified) in one or more tests. Longitudinal clinical data were not available for determination of ultimate diagnoses, including whether or not each patient met the diagnostic criteria for NTM disease as defined by the American Thoracic Society and the Infectious Disease Society of America (30). For patients with multiple NTM-positive tests, the record at the time of the first positive test was used for the analyses. For patients without an NTM-positive test result (i.e., NTM-negative patients), the record at the time of one NTM-negative test result per patient was randomly selected and used for the analyses.

Immunocompromising conditions and structural lung diseases were defined as predisposing conditions for NTM infection (2, 31 – 34), and diagnosis codes for these predisposing conditions were identified from the ICD9 and ICD10 codes (Table S1). A patient was considered predisposed to NTM infection if their one positive or randomly selected negative test record used for the analyses (as described above) was from a test performed 1 year prior to or up to 2 years following a year in which the patient received a diagnosis corresponding to one of the defined risk factors.

### Municipal water treatment.

Information on municipal water treatment practices, including disinfectant and primary source water type, for 140 cities in Michigan was obtained through two Freedom of Information Act (FOIA) requests (EPA-HQ-2015-001745 and EPA-HQ-2015-009061) communicated to the U.S. EPA (Table S2) or by reviewing a city’s annual water quality reports. The information provided by the U.S. EPA in response to our FOIA requests came from the Safe Drinking Water Information System. Four cities reported in their annual water quality reports that they blend surface and groundwater supplies. Those four cities were assigned the source water type corresponding to the source that provided the majority of the water. To identify patients residing in the cities with known water treatment practices, test record-associated addresses were passed to the Google Maps application programming interface (API) for geocoding through R. Shapefiles for cities in Michigan were accessed via the ArcGIS Online Open Data Portal maintained by the state of Michigan.

### Exclusion criteria.

Duplicate test records, records with missing or unknown test results, and test results positive for Mycobacterium tuberculosis complex were excluded (Fig. 1). Single positive cultures of Mycobacterium gordonae from any site and all M. gordonae cultures from an unknown site were excluded as likely representing clinically insignificant sample contamination. Records with addresses located in the city of Flint from 2014 to 2015 (35), and records from the city of East Lansing in 2000 (C. Dugan, personal communication, 3 August 2015) were excluded due to changes in drinking water disinfection practices in those cities during those time frames. Driving distance between each test record-associated address and Michigan Medicine (1500 E. Medical Center Drive, Ann Arbor, MI) was determined using the R package gmapsdistance (36). We excluded test records with addresses located greater than 80 miles (128.75 km) driving distance from Michigan Medicine.

### City characteristics.

U.S. 2010 demographic data (population density, percent white, and percent over 65 years old) were collected from the U.S. Census Bureau. Median household income data for 2015 for each city were supplied by U.S. Census Bureau’s American FactFinder.

### Statistical analyses.

A logistic regression model was fitted to examine the relationships between patient and city variables (including municipal drinking water variables) and an outcome of an NTM-positive test result. All statistical analyses were conducted in R (37) using α = 0.05. Covariates in the model included each patient’s age and sex, presence of a predisposing condition for NTM infection, year of test record, and driving distance from each patient’s home address to Michigan Medicine. For municipal drinking water treatment analyses, covariates included our primary variable of interest, disinfectant type (chlorine or monochloramine), as well as type of source water (groundwater or surface water) used for drinking water production. City covariates included in the model were median household income, percent individuals over 65 years old, percent individuals white, and population density. Patient age was treated as a continuous variable, and an interaction between patient age and predisposition was included in the model. Fitting a mixed-effects logistic regression model that included a random intercept for city did not offer an improvement over the logistic regression model (as determined by the AIC) and was rejected (results not shown).

The propensity score for monochloramine disinfection for each patient was estimated by a logistic regression model predicting monochloramine disinfection based upon the city and patient-level predictors in the full model (38). Following propensity score generation, inverse propensity score weights were calculated (39, 40). These weights upweight patients in one disinfectant group who, based on their propensity scores, look similar to patients in the other disinfectant group. We refitted the logistic regression model with inverse propensity score weighted data.

### Data availability.

The R code used for the analysis is available on GitHub at https://github.com/caverlyl/MI_NTM_water_epi. As the data set contains city-level identifiers of the test records, it contains protected health information and potentially identifying participant information. Thus, the data set cannot be made publicly available.
