# Supplementary material for: Retrospective Analysis of Nontuberculous Mycobacterial Infection and Monochloramine Disinfection of Municipal Drinking Water in Michigan
Source: mSphere. 2019 Jul 3;4(4):e00160-19. doi: 10.1128/mSphere.00160-19 (PMC6609225; doi:10.1128/mSphere.00160-19)
Supplement: TABLE S1 [file mSphere.00160-19-st001.docx]

| **ICD code** | **Classification** | **Code System** |
| --- | --- | --- |
| B20 | human immunodeficiency virus [HIV] disease | ICD-10 |
| B59 | pneumocystosis | ICD-10 |
| C802 | malignant neoplasm associated with transplanted organ | ICD-10 |
| C888 | other malignant immunoproliferative diseases | ICD-10 |
| C9440 | acute panmyelosis with myelofibrosis not having achieved remission | ICD-10 |
| C9441 | acute panmyelosis with myelofibrosis, in remission | ICD-10 |
| C9442 | acute panmyelosis with myelofibrosis, in relapse | ICD-10 |
| C946 | myelodysplastic disease, not classified | ICD-10 |
| D4622 | refractory anemia with excess of blasts 2 | ICD-10 |
| D471 | chronic myeloproliferative disease | ICD-10 |
| D479 | neoplasm of uncertain behavior of lymphoid, hematopoietic and related tissue, unspecified | ICD-10 |
| D47Z1 | post-transplant lymphoproliferative disorder (ptld) | ICD-10 |
| D47Z9 | other specified neoplasms of uncertain behavior of lymphoid, hematopoietic and related tissue | ICD-10 |
| D6109 | other constitutional aplastic anemia | ICD-10 |
| D61810 | antineoplastic chemotherapy induced pancytopenia | ICD-10 |
| D61811 | other drug-induced pancytopenia | ICD-10 |
| D61818 | other pancytopenia | ICD-10 |
| D700 | congenital agranulocytosis | ICD-10 |
| D701 | agranulocytosis secondary to cancer chemotherapy | ICD-10 |
| D702 | other drug-induced agranulocytosis | ICD-10 |
| D704 | cyclic neutropenia | ICD-10 |
| D708 | other neutropenia | ICD-10 |
| D709 | neutropenia, unspecified | ICD-10 |
| D71 | functional disorders of polymorphonuclear neutrophils | ICD-10 |
| D720 | genetic anomalies of leukocytes | ICD-10 |
| D72810 | lymphocytopenia | ICD-10 |
| D72818 | other decreased white blood cell count | ICD-10 |
| D72819 | decreased white blood cell count, unspecified | ICD-10 |
| D7381 | neutropenic splenomegaly | ICD-10 |
| D7581 | myelofibrosis | ICD-10 |
| D761 | hemophagocytic lymphohistiocytosis | ICD-10 |
| D762 | hemophagocytic syndrome, infection-associated | ICD-10 |
| D763 | other histiocytosis syndromes | ICD-10 |
| D800 | hereditary hypogammaglobulinemia | ICD-10 |
| D801 | nonfamilial hypogammaglobulinemia | ICD-10 |
| D802 | selective deficiency of immunoglobulin a [iga] | ICD-10 |
| D803 | selective deficiency of immunoglobulin g [igg] subclasses | ICD-10 |
| D804 | selective deficiency of immunoglobulin m [igm] | ICD-10 |
| D805 | immunodeficiency with increased immunoglobulin m [igm] | ICD-10 |
| D806 | antibody deficiency with near-normal immunoglobulins or with hyperimmunoglobulinemia | ICD-10 |
| D807 | transient hypogammaglobulinemia of infancy | ICD-10 |
| D808 | other immunodeficiencies with predominantly antibody defects | ICD-10 |
| D809 | immunodeficiency with predominantly antibody defects, unspecified | ICD-10 |
| D810 | severe combined immunodeficiency [scid] with reticular dysgenesis | ICD-10 |
| D811 | severe combined immunodeficiency [scid] with low t- and b-cell numbers | ICD-10 |
| D812 | severe combined immunodeficiency [scid] with low or normal b-cell numbers | ICD-10 |
| D814 | nezelofs syndrome | ICD-10 |
| D816 | major histocompatibility complex class i deficiency | ICD-10 |
| D817 | major histocompatibility complex class ii deficiency | ICD-10 |
| D8189 | other combined immunodeficiencies | ICD-10 |
| D819 | combined immunodeficiency, unspecified | ICD-10 |
| D820 | wiskott-aldrich syndrome | ICD-10 |
| D821 | di georges syndrome | ICD-10 |
| D822 | immunodeficiency with short-limbed stature | ICD-10 |
| D823 | immunodeficiency following hereditary defective response to epstein-barr virus | ICD-10 |
| D824 | hyperimmunoglobulin e [ige] syndrome | ICD-10 |
| D828 | immunodeficiency associated with other specified major defects | ICD-10 |
| D829 | immunodeficiency associated with major defect, unspecified | ICD-10 |
| D830 | common variable immunodeficiency with predominant abnormalities of b-cell numbers and function | ICD-10 |
| D831 | common variable immunodeficiency with predominant immunoregulatory t-cell disorders | ICD-10 |
| D832 | common variable immunodeficiency with autoantibodies to b- or t-cells | ICD-10 |
| D838 | other common variable immunodeficiencies | ICD-10 |
| D839 | common variable immunodeficiency, unspecified | ICD-10 |
| D840 | lymphocyte function antigen-1 [lfa-1] defect | ICD-10 |
| D841 | defects in the complement system | ICD-10 |
| D848 | other specified immunodeficiencies | ICD-10 |
| D849 | immunodeficiency, unspecified | ICD-10 |
| D893 | immune reconstitution syndrome | ICD-10 |
| D89810 | acute graft-versus-host disease | ICD-10 |
| D89811 | chronic graft-versus-host disease | ICD-10 |
| D89812 | acute on chronic graft-versus-host disease | ICD-10 |
| D89813 | graft-versus-host disease, unspecified | ICD-10 |
| D8982 | autoimmune lymphoproliferative syndrome [alps] | ICD-10 |
| D8989 | other specified disorders involving the immune mechanism, not elsewhere classified | ICD-10 |
| D899 | disorder involving the immune mechanism, unspecified | ICD-10 |
| E40 | kwashiorkor | ICD-10 |
| E41 | nutritional marasmus | ICD-10 |
| E42 | marasmic kwashiorkor | ICD-10 |
| E43 | unspecified severe protein-calorie malnutrition | ICD-10 |
| I120 | hypertensive chronic kidney disease with stage 5 chronic kidney disease or end stage renal disease | ICD-10 |
| I1311 | hypertensive heart and chronic kidney disease without heart failure, with stage 5 chronic kidney disease, or end stage renal disease | ICD-10 |
| I132 | hypertensive heart and chronic kidney disease with heart failure and with stage 5 chronic kidney disease, or end stage renal disease | ICD-10 |
| K912 | postsurgical malabsorption, not elsewhere classified | ICD-10 |
| M359 | systemic involvement of connective tissue, unspecified | ICD-10 |
| N185 | chronic kidney disease, stage 5 | ICD-10 |
| N186 | end stage renal disease | ICD-10 |
| T8600 | unspecified complication of bone marrow transplant | ICD-10 |
| T8601 | bone marrow transplant rejection | ICD-10 |
| T8602 | bone marrow transplant failure | ICD-10 |
| T8603 | bone marrow transplant infection | ICD-10 |
| T8609 | other complications of bone marrow transplant | ICD-10 |
| T8610 | unspecified complication of kidney transplant | ICD-10 |
| T8611 | kidney transplant rejection | ICD-10 |
| T8612 | kidney transplant failure | ICD-10 |
| T8613 | kidney transplant infection | ICD-10 |
| T8619 | other complication of kidney transplant | ICD-10 |
| T8620 | unspecified complication of heart transplant | ICD-10 |
| T8621 | heart transplant rejection | ICD-10 |
| T8622 | heart transplant failure | ICD-10 |
| T8623 | heart transplant infection | ICD-10 |
| T86290 | cardiac allograft vasculopathy | ICD-10 |
| T86298 | other complications of heart transplant | ICD-10 |
| T8630 | unspecified complication of heart-lung transplant | ICD-10 |
| T8631 | heart-lung transplant rejection | ICD-10 |
| T8632 | heart-lung transplant failure | ICD-10 |
| T8633 | heart-lung transplant infection | ICD-10 |
| T8639 | other complications of heart-lung transplant | ICD-10 |
| T8640 | unspecified complication of liver transplant | ICD-10 |
| T8641 | liver transplant rejection | ICD-10 |
| T8642 | liver transplant failure | ICD-10 |
| T8643 | liver transplant infection | ICD-10 |
| T8649 | other complications of liver transplant | ICD-10 |
| T865 | complications of stem cell transplant | ICD-10 |
| T86810 | lung transplant rejection | ICD-10 |
| T86811 | lung transplant failure | ICD-10 |
| T86812 | lung transplant infection | ICD-10 |
| T86818 | other complications of lung transplant | ICD-10 |
| T86819 | unspecified complication of lung transplant | ICD-10 |
| T86830 | bone graft rejection | ICD-10 |
| T86831 | bone graft failure | ICD-10 |
| T86832 | bone graft infection | ICD-10 |
| T86838 | other complications of bone graft | ICD-10 |
| T86839 | unspecified complication of bone graft | ICD-10 |
| T86850 | intestine transplant rejection | ICD-10 |
| T86851 | intestine transplant failure | ICD-10 |
| T86852 | intestine transplant infection | ICD-10 |
| T86858 | other complications of intestine transplant | ICD-10 |
| T86859 | unspecified complication of intestine transplant | ICD-10 |
| T86890 | other transplanted tissue rejection | ICD-10 |
| T86891 | other transplanted tissue failure | ICD-10 |
| T86892 | other transplanted tissue infection | ICD-10 |
| T86898 | other complications of other transplanted tissue | ICD-10 |
| T86899 | unspecified complication of other transplanted tissue | ICD-10 |
| T8690 | unspecified complication of unspecified transplanted organ and tissue | ICD-10 |
| T8691 | unspecified transplanted organ and tissue rejection | ICD-10 |
| T8692 | unspecified transplanted organ and tissue failure | ICD-10 |
| T8693 | unspecified transplanted organ and tissue infection | ICD-10 |
| T8699 | other complications of unspecified transplanted organ and tissue | ICD-10 |
| Z4821 | encounter for aftercare following heart transplant | ICD-10 |
| Z4822 | encounter for aftercare following kidney transplant | ICD-10 |
| Z4823 | encounter for aftercare following liver transplant | ICD-10 |
| Z4824 | encounter for aftercare following lung transplant | ICD-10 |
| Z48280 | encounter for aftercare following heartlung transplant | ICD-10 |
| Z48290 | encounter for aftercare following bone marrow transplant | ICD-10 |
| Z48298 | encounter for aftercare following other organ transplant | ICD-10 |
| Z4901 | encounter for fitting and adjustment of extracorporeal dialysis catheter | ICD-10 |
| Z4902 | encounter for fitting and adjustment of peritoneal dialysis catheter | ICD-10 |
| Z4931 | encounter for adequacy testing for hemodialysis | ICD-10 |
| Z940 | kidney transplant status | ICD-10 |
| Z941 | heart transplant status | ICD-10 |
| Z942 | lung transplant status | ICD-10 |
| Z943 | heart and lungs transplant status | ICD-10 |
| Z944 | liver transplant status | ICD-10 |
| Z9481 | bone marrow transplant status | ICD-10 |
| Z9482 | intestine transplant status | ICD-10 |
| Z9483 | pancreas transplant status | ICD-10 |
| Z9484 | stem cells transplant status | ICD-10 |
| Z9489 | other transplanted organ and tissue status | ICD-10 |
| Z992 | dependence on renal dialysis | ICD-10 |
| E84.0 | cystic fibrosis with pulmonary manifestations | ICD-10 |
| E84.1 | cystic fibrosis with intestinal manifestations | ICD-10 |
| E84.11 | meconium ileus in cystic fibrosis | ICD-10 |
| E84.19 | cystic fibrosis with other intestinal manifestations | ICD-10 |
| E84.8 | cystic fibrosis with other manifestations | ICD-10 |
| E84.9 | cystic fibrosis, unspecified | ICD-10 |
| E88.01 | alpha-1-antitrypsin deficiency | ICD-10 |
| J44 | other chronic obstructive pulmonary disease | ICD-10 |
| J44.0 | chronic obstructive pulmonary disease with acute lower respiratory infection | ICD-10 |
| J44.1 | chronic obstructive pulmonary disease with (acute) exacerbation | ICD-10 |
| J44.9 | chronic obstructive pulmonary disease, unspecified | ICD-10 |
| J47.0 | bronchiectasis with acute lower respiratory infection | ICD-10 |
| J47.1 | bronchiectasis with (acute) exacerbation | ICD-10 |
| J47.9 | bronchiectasis, uncomplicated | ICD-10 |
| J60 | coalworker's pneumoconiosis | ICD-10 |
| J61 | pneumoconiosis due to asbestos and other mineral fibers | ICD-10 |
| J62 | pneumoconiosis due to dust containing silica | ICD-10 |
| J63 | pneumoconiosis due to other inorganic dusts | ICD-10 |
| J64 | unspecified pneumoconiosis | ICD-10 |
| J65 | pneumoconiosis associated with tuberculosis | ICD-10 |
| J84.01 | alveolar proteinosis | ICD-10 |
| Q89.3 | kartagener’s syndrome of triad (sinusitis, bronchiectasis, situs inversus) | ICD-10 |
| J40 | bronchitis, not specified as acute or chronic | ICD-10 |
| J41 | simple and mucopurulent chronic bronchitis | ICD-10 |
| J42 | unspecified chronic bronchitis | ICD-10 |
| J43 | emphysema | ICD-10 |
| J44 | other chronic obstructive pulmonary disease | ICD-10 |
| J47 | bronchiectasis | ICD-10 |
| 42 | human immunodeficiency virus | ICD-9 |
| 1363 | pneumocystosis | ICD-9 |
| 1992 | malignant neoplasm associated with transplanted organ (oct08) | ICD-9 |
| 23873 | hi grde myelodys syn les (oct06) | ICD-9 |
| 23876 | myelofi w myelo metaplas (oct06) | ICD-9 |
| 23877 | neoplasm of uncertain behavior, post-transplant lymphoproliferative disorder (ptld) (oct08) | ICD-9 |
| 23879 | neoplasm of uncertain behavior, other lymphatic and hematopoietic tissues (oct08) | ICD-9 |
| 260 | kwashiorkor (oct05) | ICD-9 |
| 261 | nutritional marasmus (oct05) | ICD-9 |
| 262 | oth severe malnutrition (oct05) | ICD-9 |
| 27900 | hypogammaglobulinem nos | ICD-9 |
| 27901 | selective iga immunodef | ICD-9 |
| 27902 | selective igm immunodef | ICD-9 |
| 27903 | selective ig defic nec | ICD-9 |
| 27904 | cong hypogammaglobulinem | ICD-9 |
| 27905 | immunodefic w hyper-igm | ICD-9 |
| 27906 | common variabl immunodef | ICD-9 |
| 27909 | humoral immunity def nec | ICD-9 |
| 27910 | immundef t-cell def nos | ICD-9 |
| 27911 | digeorges syndrome | ICD-9 |
| 27912 | wiskott-aldrich syndrome | ICD-9 |
| 27913 | nezelofs syndrome | ICD-9 |
| 27919 | defic cell immunity nos | ICD-9 |
| 2792 | combined immunity deficiency | ICD-9 |
| 2793 | unspecified immunity deficiency | ICD-9 |
| 2794 | autoimmune disease, not elsewhere classified | ICD-9 |
| 27941 | autoimmune lymphoproliferative syndrome alps (oct09) | ICD-9 |
| 27949 | autoimmune disease, not elsewhere classified (oct09) | ICD-9 |
| 27950 | graft-versus-host disease unspecified (oct08) | ICD-9 |
| 27951 | acute graft-versus-host disease (oct08) | ICD-9 |
| 27952 | chronic graft-versus-host disease (oct08) | ICD-9 |
| 27953 | acute on chronic graft-versushost disease (oct08) | ICD-9 |
| 2798 | other specified disorders involving the immune mechanism | ICD-9 |
| 2799 | unspecified disorder of immune mechanism | ICD-9 |
| 28409 | const aplastc anemia nec (oct06) | ICD-9 |
| 2841 | pancytopenia (oct06) | ICD-9 |
| 2880 | agranulocytosis (oct05) | ICD-9 |
| 28800 | neutropenia nos (oct06) | ICD-9 |
| 28801 | congenital neutropenia (oct06) | ICD-9 |
| 28802 | cyclic neutropenia (oct06) | ICD-9 |
| 28803 | drug induced neutropenia (oct06) | ICD-9 |
| 28809 | neutropenia nec (oct06) | ICD-9 |
| 2882 | genetic anomaly leukocyt (oct06) | ICD-9 |
| 2884 | hemophagocytic syndromes | ICD-9 |
| 28850 | leukocytopenia nos (oct06) | ICD-9 |
| 28851 | lymphocytopenia (oct06) | ICD-9 |
| 28859 | decreased wbc count nec (oct06 | ICD-9 |
| 28953 | neutropenic splenomegaly | ICD-9 |
| 28983 | myelofibrosis (oct06) | ICD-9 |
| 40301 | mal hyp kidney w chr kid (oct06) | ICD-9 |
| 40311 | ben hyp kidney w chr kid (oct06) | ICD-9 |
| 40391 | hyp kidney nos w chr kid (oct06) | ICD-9 |
| 40402 | mal hy hrt/kid w chr kid (oct06) | ICD-9 |
| 40403 | mal hyp hrt/kid w hf/kid (oct06) | ICD-9 |
| 40412 | ben hyp ht/kid w chr kid (oct06) | ICD-9 |
| 40413 | ben hyp ht/kid w hf/kid (oct06) | ICD-9 |
| 40492 | hyp ht/kid nos w chr kid (oct06) | ICD-9 |
| 40493 | hyp hrt/kid nos w hf/kid (oct06) | ICD-9 |
| 5793 | intest postop nonabsorb (oct06) | ICD-9 |
| 585 | hronic kidney disease (oct05) | ICD-9 |
| 5855 | chron kidney dis stage v (oct05) | ICD-9 |
| 5856 | end stage renal disease (oct06) | ICD-9 |
| 9968 | complications of transplanted organ | ICD-9 |
| 99680 | comp organ transplnt nos | ICD-9 |
| 99681 | compl kidney transplant | ICD-9 |
| 99682 | compl liver transplant | ICD-9 |
| 99683 | compl heart transplant | ICD-9 |
| 99684 | compl lung transplant | ICD-9 |
| 99685 | compl marrow transplant | ICD-9 |
| 99686 | compl pancreas transplnt | ICD-9 |
| 99687 | comp intestine transplnt | ICD-9 |
| 99689 | comp oth organ transplnt | ICD-9 |
| V420 | kidney replaced by transplant | ICD-9 |
| V421 | heart replaced by transplant | ICD-9 |
| V426 | lung replaced by transplant | ICD-9 |
| V427 | liver replaced by transplant | ICD-9 |
| V428 | other specified organ or tissue | ICD-9 |
| V4281 | bone marrow specified by transplant | ICD-9 |
| V4282 | peripheral stem cells replaced by transplant | ICD-9 |
| V4283 | pancreas replaced by transplant | ICD-9 |
| V4284 | intestines replace by transplant | ICD-9 |
| V4289 | other replaced by transplant | ICD-9 |
| V451 | renal dialysis status (oct06) | ICD-9 |
| V4511 | renal dialysis status (oct08) | ICD-9 |
| V560 | renal dialysis encounter (oct06) | ICD-9 |
| V561 | ft/adj xtrcorp dial cath (oct06) | ICD-9 |
| V562 | fit/adj perit dial cath (oct06) | ICD-9 |
| 18 | infus immunosup antibody (oct05) | ICD-9 |
| 335 | lung transplantation | ICD-9 |
| 3350 | lung transplantation, nos | ICD-9 |
| 3351 | unilateral lung transplantation | ICD-9 |
| 3352 | bilateral lung transplantation | ICD-9 |
| 336 | combined heart-lung transplantation | ICD-9 |
| 375 | heart transplantation | ICD-9 |
| 3751 | heart transplantation (oct03) | ICD-9 |
| 410 | operations on bone marrow and spleen | ICD-9 |
| 4100 | bone marrow transplant, nos | ICD-9 |
| 4101 | autologous bone marrow transplant w/o purging | ICD-9 |
| 4102 | allogeneic bone marrow transplant w/ purging | ICD-9 |
| 4103 | allogeneic bone marrow transplant w/o purging | ICD-9 |
| 4104 | autologous hematopoietic stem cell transplant w/o purging | ICD-9 |
| 4105 | allogeneic hematopoietic stem cell transplant w/o purging | ICD-9 |
| 4106 | cord blood stem cell transplant | ICD-9 |
| 4107 | autologous hematopoietic stem cell transplant w/ purging | ICD-9 |
| 4108 | allogeneic hematopoietic stem cell transplant w/ purging | ICD-9 |
| 4109 | autologous bone marrow transplant w/ purging | ICD-9 |
| 5051 | auxiliary liver transplant | ICD-9 |
| 5059 | liver transplant, nec | ICD-9 |
| 5280 | pancreatic transplant, nos | ICD-9 |
| 5281 | reimplantation of pancreatic tissue | ICD-9 |
| 5282 | homotransplant of pancreas | ICD-9 |
| 5283 | heterotransplant of pancreas | ICD-9 |
| 5285 | allotransplantation of cells of islets of langerhans | ICD-9 |
| 5286 | transplantation of cells of islets of langerhans, nos | ICD-9 |
| 5569 | other kidney transplantation | ICD-9 |
| 759.3 | kartagener’s syndrome of triad (sinusitis, bronchiectasis, situs inversus) | ICD-9 |
| 277 | cystic fibrosis without mention of meconium ileus | ICD-9 |
| 277.01 | cystic fibrosis with meconium ileus | ICD-9 |
| 277.02 | cystic fibrosis with pulmonary manifestations | ICD-9 |
| 277.03 | cystic fibrosis with gastrointestinal manifestations | ICD-9 |
| 277.09 | cystic fibrosis with other manifestations | ICD-9 |
| 273.4 | alpha-1-antitrypsin deficiency | ICD-9 |
| 494 | bronchiectasis without acute exacerbation | ICD-9 |
| 494.1 | bronchiectasis with acute exacerbation | ICD-9 |
| 500 | coal workers' pneumoconiosis | ICD-9 |
| 502 | pneumoconiosis due to other silica or silicates | ICD-9 |
| 503 | pneumoconiosis due to other inorganic dust | ICD-9 |
| 505 | pneumoconiosis, unspecified | ICD-9 |
| 516 | pulmonary alveolar proteinosis | ICD-9 |
| 490 | bronchitis, not specified as acute or chronic | ICD-9 |
| 491 | simple chronic bronchitis | ICD-9 |
| 491.1 | mucopurulent chronic bronchitis | ICD-9 |
| 491.2 | obstructive chronic bronchitis | ICD-9 |
| 491.2 | obstructive chronic bronchitis without exacerbation | ICD-9 |
| 491.21 | obstructive chronic bronchitis with (acute) exacerbation | ICD-9 |
| 491.22 | obstructive chronic bronchitis with acute bronchitis | ICD-9 |
| 491.8 | other chronic bronchitis | ICD-9 |
| 491.9 | unspecified chronic bronchitis | ICD-9 |
| 492 | emphysematous bleb | ICD-9 |
| 492.8 | other emphysema | ICD-9 |
| 494 | bronchiectasis without acute exacerbation | ICD-9 |
| 494.1 | bronchiectasis with acute exacerbation | ICD-9 |
| 495 | farmers' lung | ICD-9 |
| 495.1 | bagassosis | ICD-9 |
| 495.2 | bird-fanciers' lung | ICD-9 |
| 495.3 | suberosis | ICD-9 |
| 495.4 | malt workers' lung | ICD-9 |
| 495.5 | mushroom workers' lung | ICD-9 |
| 495.6 | maple bark-strippers' lung | ICD-9 |
| 495.7 | ventilation\pneumonitis | ICD-9 |
| 495.8 | other specified allergic alveolitis and pneumonitis | ICD-9 |
| 495.9 | unspecified allergic alveolitis and pneumonitis | ICD-9 |
| 496 | chronic airway obstruction, not elsewhere classified | ICD-9 |
