# Supplementary material for: Retrospective Analysis of Nontuberculous Mycobacterial Infection and Monochloramine Disinfection of Municipal Drinking Water in Michigan
Source: mSphere. 2019 Jul 3;4(4):e00160-19. doi: 10.1128/mSphere.00160-19 (PMC6609225; doi:10.1128/mSphere.00160-19)
Supplement: TABLE S2 [file mSphere.00160-19-st002.docx]

|  | **City Name** | **Source water***^a^* | **Disinfectant** |
| --- | --- | --- | --- |
| 1 | Adrian | SW | Chlorine |
| 2 | Albion | GW | Chlorine |
| 3 | Allen Park | SW | Chlorine |
| 4 | Alma | GW | Chlorine |
| 5 | Alpena | SW | Chlorine |
| 6 | Ann Arbor | SW | Monochloramine |
| 7 | Auburn Hills | SW | Chlorine |
| 8 | Battle Creek | GW | Chlorine |
| 9 | Bay City | SW | Chlorine |
| 10 | Belding | GW | Chlorine |
| 11 | Belleville | SW | Chlorine |
| 12 | Benton Harbor | SW | Chlorine |
| 13 | Berkley | SW | Chlorine |
| 14 | Birmingham | SW | Chlorine |
| 15 | Bloomfield Hills | SW | Chlorine |
| 16 | Brighton | GW | Chlorine |
| 17 | Buchanan | GW | Chlorine |
| 18 | Burton | SW | Chlorine |
| 19 | Cadillac | GW | Chlorine |
| 20 | Carson City | GW | Chlorine |
| 21 | Center Line | SW | Chlorine |
| 22 | Charlevoix | GW | Chlorine |
| 23 | Charlotte | GW | Chlorine |
| 24 | Cheboygan | GW | Chlorine |
| 25 | Chelsea | GW | Chlorine |
| 26 | Clare | GW | Chlorine |
| 27 | Clawson | SW | Chlorine |
| 28 | Coldwater | GW | Chlorine |
| 29 | Croswell | GW | Chlorine |
| 30 | Dearborn | SW | Chlorine |
| 31 | Dearborn Heights | SW | Chlorine |
| 32 | Detroit | SW | Chlorine |
| 33 | Dexter | GW | Chlorine |
| 34 | Durand | GW | Chlorine |
| 35 | East Lansing | GW | Monochloramine |
| 36 | Eastpointe | SW | Chlorine |
| 37 | Ecorse | SW | Chlorine |
| 38 | Escanaba | SW | Chlorine |
| 39 | Essexville | SW | Chlorine |
| 40 | Farmington | SW | Chlorine |
| 41 | Farmington Hills | SW | Chlorine |
| 42 | Fenton | GW | Chlorine |
| 43 | Ferndale | SW | Chlorine |
| 44 | Flat Rock | SW | Chlorine |
| 45 | Flint | SW | Chlorine |
| 46 | Flushing | SW | Chlorine |
| 47 | Fraser | SW | Chlorine |
| 48 | Garden City | SW | Chlorine |
| 49 | Gibraltar | SW | Chlorine |
| 50 | Gladstone | SW | Chlorine |
| 51 | Grand Blanc | GW | Chlorine |
| 52 | Grand Ledge | GW | Chlorine |
| 53 | Grand Rapids | SW | Chlorine |
| 54 | Grosse Pointe | SW | Chlorine |
| 55 | Grosse Pointe Farms | SW | Chlorine |
| 56 | Grosse Pointe Park | SW | Chlorine |
| 57 | Grosse Pointe Woods | SW | Chlorine |
| 58 | Hamtramck | SW | Chlorine |
| 59 | Harbor Beach | SW | Chlorine |
| 60 | Harper Woods | SW | Chlorine |
| 61 | Hastings | GW | Chlorine |
| 62 | Hazel Park | SW | Chlorine |
| 63 | Highland Park | SW | Chlorine |
| 64 | Hillsdale | GW | Chlorine |
| 65 | Howell | GW | Chlorine |
| 66 | Huntington Woods | SW | Chlorine |
| 67 | Inkster | SW | Chlorine |
| 68 | Jackson | GW | Chlorine |
| 69 | Kalamazoo | GW | Chlorine |
| 70 | Keego Harbor | SW | Chlorine |
| 71 | Lake Angelus | SW | Chlorine |
| 72 | Lansing | GW | Monochloramine |
| 73 | Lapeer | SW | Chlorine |
| 74 | Lathrup Village | SW | Chlorine |
| 75 | Lincoln Park | SW | Chlorine |
| 76 | Linden | GW | Chlorine |
| 77 | Livonia | SW | Chlorine |
| 78 | Lowell | GW | Chlorine |
| 79 | Madison Heights | SW | Chlorine |
| 80 | Manistique | SW | Chlorine |
| 81 | Marshall | GW | Chlorine |
| 82 | Melvindale | SW | Chlorine |
| 83 | Memphis | SW | Chlorine |
| 84 | Menominee | SW | Chlorine |
| 85 | Midland | SW | Chlorine |
| 86 | Milan | GW | Chlorine |
| 87 | Monroe | SW | Chlorine |
| 88 | Morenci | GW | Chlorine |
| 89 | Mt Clemens | SW | Chlorine |
| 90 | Mt Pleasant | GW | Chlorine |
| 91 | Muskegon | SW | Chlorine |
| 92 | New Baltimore | SW | Chlorine |
| 93 | New Buffalo | SW | Chlorine |
| 94 | Niles | GW | Chlorine |
| 95 | Northville | SW | Chlorine |
| 96 | Novi | SW | Chlorine |
| 97 | Oak Park | SW | Chlorine |
| 98 | Orchard Lake Village | SW | Chlorine |
| 99 | Owosso | GW | Chlorine |
| 100 | Petoskey | GW | Chlorine |
| 101 | Pleasant Ridge | SW | Chlorine |
| 102 | Plymouth | SW | Chlorine |
| 103 | Pontiac | SW | Chlorine |
| 104 | Port Huron | SW | Chlorine |
| 105 | Portage | GW | Chlorine |
| 106 | River Rouge | SW | Chlorine |
| 107 | Riverview | SW | Chlorine |
| 108 | Rochester Hills | SW | Chlorine |
| 109 | Rockwood | SW | Chlorine |
| 110 | Romulus | SW | Chlorine |
| 111 | Roseville | SW | Chlorine |
| 112 | Royal Oak | SW | Chlorine |
| 113 | Saginaw | SW | Chlorine |
| 114 | Saline | GW | Chlorine |
| 115 | Sault Ste Marie | SW | Chlorine |
| 116 | South Lyon | GW | Chlorine |
| 117 | Southfield | SW | Chlorine |
| 118 | Southgate | SW | Chlorine |
| 119 | St Clair Shores | SW | Chlorine |
| 120 | St Ignace | SW | Chlorine |
| 121 | St Joseph | SW | Chlorine |
| 122 | Sterling Heights | SW | Chlorine |
| 123 | Sturgis | GW | Chlorine |
| 124 | Sylvan Lake | SW | Chlorine |
| 125 | Taylor | SW | Chlorine |
| 126 | Tecumseh | GW | Chlorine |
| 127 | The Village of Grosse Pointe Shores A Michigan City | SW | Chlorine |
| 128 | Trenton | SW | Chlorine |
| 129 | Troy | SW | Chlorine |
| 130 | Utica | SW | Chlorine |
| 131 | Village of Clarkston | GW | Chlorine |
| 132 | Walled Lake | SW | Chlorine |
| 133 | Warren | SW | Chlorine |
| 134 | Wayne | SW | Chlorine |
| 135 | Westland | SW | Chlorine |
| 136 | Wixom | SW | Chlorine |
| 137 | Woodhaven | SW | Chlorine |
| 138 | Wyandotte | SW | Chlorine |
| 139 | Wyoming | SW | Chlorine |
| 140 | Ypsilanti | SW | Chlorine |

*^a^*Four cities (Ann Arbor, Adrian, Alma and Mt. Pleasant) reported in their annual water quality reports that they blend surface and groundwater supplies.
