# Supplementary material for: Retrospective Analysis of Nontuberculous Mycobacterial Infection and Monochloramine Disinfection of Municipal Drinking Water in Michigan
Source: mSphere. 2019 Jul 3;4(4):e00160-19. doi: 10.1128/mSphere.00160-19 (PMC6609225; doi:10.1128/mSphere.00160-19)
Supplement: TABLE S3 [file mSphere.00160-19-st003.docx]

|  | Unweighted | | Weighted | |
| --- | --- | --- | --- | --- |
|  | Difference | *p* | Difference | *p* |
| Sex (male) | 4.1% | <0.001 | 3.7% | <0.01 |
| Age (years) | 2.47 | <0.001 | 1.98 | <0.001 |
| Predisposed | 10.1% | <0.001 | 9.1% | <0.001 |
| Sample year | 0.02 | >0.05 | 0.02 | >0.05 |
| Driving distance to Michigan Medicine (mi) | 24.8 | <0.001 | 22.1 | <0.001 |
| Population density | 673 | <0.001 | 616 | <0.001 |
| Drinking water source  (surface water) | 3.5% | <0.001 | 1.7% | >0.05 |
| Percent population older than 65 years old | 4.5% | <0.01 | 4.2% | <0.01 |
| Percent population white | 0.53% | >0.05 | 1.16% | <0.05 |
| Median income (USD) | 4720 | <0.001 | 3586 | <0.001 |
