# Supplementary material for: Retrospective Analysis of Nontuberculous Mycobacterial Infection and Monochloramine Disinfection of Municipal Drinking Water in Michigan
Source: mSphere. 2019 Jul 3;4(4):e00160-19. doi: 10.1128/mSphere.00160-19 (PMC6609225; doi:10.1128/mSphere.00160-19)
Supplement: TABLE S4 [file mSphere.00160-19-st004.docx]

| Predictor | Variable type | *p*- Value | OR (95% CI) |
| --- | --- | --- | --- |
| Sex (male) | Patient | 0.07 | 0.84 (0.70-1.01) |
| Age (years) | Patient | < 0.001 | 1.02 (1.01-1.02) |
| Predisposed | Patient | < 0.001 | 7.60 (4.51-12.88) |
| Interaction between age and predisposing condition | Patient | < 0.001 | 0.98 (0.97-0.99) |
| Sample year | Patient | 0.04 | 0.98 (0.96-1.00) |
| Driving distance to Michigan Medicine | Patient | 0.43 | 1.00 (1.00-1.00) |
| Population density | City | 0.001 | 1.00 (1.00-1.00) |
| Drinking water source (surface water) | City | 0.17 | 1.24 (0.92-1.69) |
| Drinking water disinfectant (monochloramine) | City | 0.49 | 1.11 (0.83-1.47) |
| Percent population older than 65 years old | City | 0.45 | 0.99 (0.95-1.02) |
| Percent population white | City | 0.08 | 1.00 (1.00-1.01) |
| Log(median income) | City | 0.20 | 1.30 (0.87-1.94) |
