# Supplementary material for: Retrospective Analysis of Nontuberculous Mycobacterial Infection and Monochloramine Disinfection of Municipal Drinking Water in Michigan
Source: mSphere. 2019 Jul 3;4(4):e00160-19. doi: 10.1128/mSphere.00160-19 (PMC6609225; doi:10.1128/mSphere.00160-19)
Supplement: TABLE S5 [file mSphere.00160-19-st005.docx]

| Category | Terms |
| --- | --- |
| Lung | BAL; BRON BX; BRONCH; BRONCH BRUSH; BRONCH L; BRONCH R; BRONCHI; BRONCHI, LEF; BRONCHI, RIG; BRONCHIAL BI; BRONCHIAL WA; BRONCHO-ALVE; BRWSH; CHEST; CHEST T; CHEST TUBE; ENDOTR T; ENDOTRAC; ENDOTRACHEAL; ENDOTRACHIAL; LEFT LUNG; LEFT LUNG, L; LEFT LUNG, U; LUNG; LUNG L; LUNG LLL; LUNG LUL; LUNG R; LUNG RLL; LUNG RUL; PLEURAL; PLEURAL FLUI; RIGHT LUNG; RIGHT LUNG,; SPUT; SPUTIND; SPUTUM; SPUTUM, INDU; THORACENTESI; THORACIC; THORFL; TRACH; TRACHASP; TRACHEAL; TRACHEAL ASP |
| Gastrointestinal | ABDFL; ABDOMEN; ABDOMINA; ABDOMINAL; ABDOMINAL FL; ASCITES; ASCITES FLUI; BILE; COLON; DUODENAL ASP; ESOPHAGUS; FECES; GALLBLADDER; GASTRIC; GASTRIC FLUI; GLBLAD; LIVER; PANCRE; PANCREAS; PARACENTESIS; PERIT; PERITON; PERITONEAL; PERITONEAL D; PERITONEAL F; RECTAL; RIGHT COLON; ULCER |
| Lymphatic | LYMPH NODE; LYNODE; NODE |
| Blood and cardiovascular | AORTIC; ARTERY; BLD; BLOOD; BONE MAR; BONE MARROW; CLOT; HEART; MITRAL V; MITRAL VALVE; PERICAR; PERICARD; PERICARDIAL; PERICARDIUM; PLASMA; SERUM; VEIN |
| Skin and musculoskeletal | ANKLE; ANKLE L; ANKLE R; ARM; ARM L; ARM R; AXILLA; BACK; BONE; BRACHIAL; BREAST; BREAST L; BREAST R; BREAST, LEFT; BREAST, RIGH; BURSA; BUTT; BUTT L; BUTTOCKS; BUTTOCKS, LE; BUTTOCKS, RI; CALF; CALF L; CALF R; CARTILAG; CARTILAGE; CHEEK L; CHEEK R; CHIN; CLAVICLE; CONJUNCTIVA; CORN; CORN DON; CORN L; CORN R; CORNEA; CORNEAL TRAN; DISC; EAR; EAR L; EAR MIDL; EAR MIDR; EAR R; ELBOW; ELBOW L; ELBOW R; EYE; EYE R; FACE; FEMORAL; FEMUR; FEMUR L; FEMUR R; FIBULA; FING; FINGER; FINGER NAIL; FLANK; FOOT; FOOT L; FOOT R; FOREARM; GINGIVA; GROIN; HAND; HAND L; HAND R; HIP; HIP L; HIP R; ILIAC CR; INDEX FINGER; INGUINAL; JAW; JOINT; JOINT FLUID; KNEE; KNEE L; KNEE R; LEFT ANKLE; LEFT ARM; LEFT CALF; LEFT CHEEK; LEFT CLAVICL; LEFT EAR; LEFT ELBOW; LEFT EYE; LEFT FEMUR; LEFT FOOT; LEFT HAND; LEFT HEEL; LEFT HIP; LEFT KNEE; LEFT LEG; LEFT PALM; LEFT SHOULDE; LEFT TOE; LEFT WRIST; LEG; LEG L; LEG R; LIGAMENT; LIP; LOWER BACK; MAND; MANDIBLE; METATARS; MIDDLE EAR F; MOUTH; MUCOSA; NAIL; NECK; OCULAR; ORBIT; PALATE; PALM L; PATELLA; PERIANAL; PERINEAL; PERINEUM; PERIRECTAL; PERITONSILLA; PHARYNX; RIB; RIGHT ANKLE; RIGHT ARM; RIGHT CHEEK; RIGHT CLAVIC; RIGHT EAR; RIGHT ELBOW; RIGHT EYE; RIGHT FEMUR; RIGHT FOOT; RIGHT HAND; RIGHT HEEL; RIGHT HIP; RIGHT KNEE; RIGHT LEG; RIGHT SHOULD; RIGHT TOE; RIGHT WRIST; SACRAL; SCALP; SHOULD; SHOULD L; SHOULDER; SKIN; STERNAL; STERNUM; SYNFL; SYNOVIAL FLU; SYNOVIUM; TENDON; THIGH; THIGH L; THIGH R; THIGH, LEFT; THIGH, RIGHT; THUMB; TIBIA; TOE; TOE R; TOENAIL; UPPER BACK; VERTEBRA; VITREOUS FLU; WOUND; WRIST; WRIST L; WRIST R |
| Sinus | ETHMOID SINU; FRONTAL SINU; LEFT MAXILLA; MASTOID; MAXILLAR; MAXILLARY; MAXILLARY SI; NASAL; NASO; NASOPHARYNX; NOSE; RIGHT MAXILL; SINUS; SINUS E; SINUS F; SINUS ML; SINUS MR |
| Central nervous system | BRAIN; CEREBROSPINA; CSF; SPINAL; VENTRICULOST |
| Genitourinary | ENDOMETRIUM; ENDOMT; KID; KIDNEY; LEFT KIDNEY; NEPHROSTOMY; OVARY; PERINEPH; PERINEPHRIC; RIGHT KIDNEY; SCROT; SCROTUM; SEMEN; TESTICLE; UR CATH; URINE; URINE, CATHE; UTERINE |
| Other | ABSCESS; ADENOID; ALLO; ALLOGRAFT; ASPIRATE; ASPIRATED SP; ASPSPT; BF; BIOPSY; BLAD; BLISTER; BODY FLUID; BRBR; BUCCAL; CATHETER TIP; CERVICAL; CYST; CYST FLUID; DIALYS; DIALYSATE; DISCHARGE; DONOR; DR; DRAIN; DRAINAGE; EPIDURAL; EXUDATE; FB; FB CATH; FETUS; FISTULA; FLUID; FOREIGN BODY; GRAFT; GUNSHOT; HEMATOMA; INCISION; JACKSON PRAT; JP; LEFT TONSIL; LESION; LINE; LOBE; LUMBAR; MASS; MAST; MUCUS; NODULE; OTHER; PACE W; PACEMAKER; PACEMAKER LE; PACEMAKER PO; PARAFL; PAROTID; PD FLUID; PELVIS; PERCUTANEOUS; PLFL; PSCP; PUS; RIGHT TONSIL; SHUNT; SPLEEN; STUMP; SUBDURAL FLU; SUPRAPUBIC; SWAB OF UNKN; TISSUE; TONSIL; TUBE; UR; VALVE; VENTRI; VENTRICULAR; WND |
